# Supplementary material for: Cost-effectiveness of avelumab first-line maintenance therapy for adult patients with locally advanced or metastatic urothelial carcinoma in France
Source: PLoS One. 2024 May 10;19(5):e0302548. doi: 10.1371/journal.pone.0302548 (PMC11086848; doi:10.1371/journal.pone.0302548)
Supplement: Attachment — Submitted filename: Response to Reviewers_GEVD France UC CE Manuscript_27Sept23.docx [file pone.0302548.s001.docx]

**Response to reviewers’ comments**

**Manuscript title:** Cost-effectiveness of avelumab first-line maintenance therapy for adult patients with locally advanced or metastatic urothelial carcinoma in France

**Manuscript ID:** PONE-D-23-16035

**Authors:** Fanny Porte, Anna Granghaud, Jane Chang, Mairead Kearney, Aya Morel, Ingrid Plessala, Hélène Cawston, Julie Roiz, Ying Xiao, Marie-Noelle Solbes, Prisca Lambert, Alain Ravaud, Yohann Loriot, Antoine Thiery-Vuillemin, Pierre Lévy

Dear Dr Chenette,

Thank you very much for providing us with the opportunity to improve our manuscript based on the thoughtful commentary from you and the peer reviewers. Our responses to the comments are provided in the table below.

| **No.** | **Journal Requirements** | **Response and proposed revisions (in red)** |
| --- | --- | --- |
| **1** | Please ensure that your manuscript meets PLOS ONE's style requirements, including those for file naming. The PLOS ONE style templates can be found at  <https://journals.plos.org/plosone/s/file?id=wjVg/PLOSOne_formatting_sample_main_body.pdf> and  <https://journals.plos.org/plosone/s/file?id=ba62/PLOSOne_formatting_sample_title_authors_affiliations.pdf> | The manuscript has been updated to ensure it meets PLOS ONE’s style requirements. |
| **2** | Thank you for stating the following financial disclosure:  “This study was funded by Merck Santé S.A.S, an affiliate of Merck KGaA, Darmstadt, Germany (CrossRef Funder ID: 10.13039/100009945), as part of an alliance between the healthcare business of Merck KGaA, Darmstadt, Germany and Pfizer.”  Please state what role the funders took in the study. If the funders had no role, please state: "The funders had no role in study design, data collection and analysis, decision to publish, or preparation of the manuscript."  If this statement is not correct you must amend it as needed.  Please include this amended Role of Funder statement in your cover letter; we will change the online submission form on your behalf. | The funding statement has been updated as follow:  “*Authors who were employees of the funders were involved in the model design, data collection, analysis, manuscript preparation, and the decision to publish*.”  We have also updated the wording to specify the end of the alliance between Merck and Pfizer since the manuscript was developed. |
| **3** | Thank you for stating the following in the Acknowledgments Section of your manuscript:  “This study was funded by Merck Santé S.A.S, an affiliate of Merck KGaA, Darmstadt, Germany (CrossRef Funder ID: 10.13039/100009945), as part of an alliance between the healthcare business of Merck KGaA, Darmstadt, Germany and Pfizer. Medical writing was provided by Paola Marino and Sudipta Ridhurkar from Amaris Consulting and editorial support was provided by Clinical Thinking, funded by Merck Santé S.A.S, an affiliate of Merck KGaA, Darmstadt, Germany, and Pfizer.”  We note that you have provided funding information that is currently declared in your Funding Statement. However, funding information should not appear in the Acknowledgments section or other areas of your manuscript. We will only publish funding information present in the Funding Statement section of the online submission form.  Please remove any funding-related text from the manuscript and let us know how you would like to update your Funding Statement. Currently, your Funding Statement reads as follows:  “This study was funded by Merck Santé S.A.S, an affiliate of Merck KGaA, Darmstadt, Germany (CrossRef Funder ID: 10.13039/100009945), as part of an alliance between the healthcare business of Merck KGaA, Darmstadt, Germany and Pfizer. Please include your amended statements within your cover letter; we will change the online submission form on your behalf.” | The Acknowledgments Section has been updated to remove any funding-related text:  *“~~This study was funded by Merck Santé S.A.S, an affiliate of Merck KGaA, Darmstadt, Germany (CrossRef Funder ID: 10.13039/100009945), as part of an alliance between the healthcare business of Merck KGaA, Darmstadt, Germany and Pfizer.~~ Medical writing was provided by Paola Marino and Sudipta Ridhurkar from Amaris Consulting and editorial support was provided by Clinical Thinking, and was funded by Merck Santé S.A.S, an affiliate of Merck KGaA, Darmstadt, Germany, and Pfizer.”* |
| **4** | Thank you for stating the following in the Competing Interests section:  “F. Porte is an employee of Merck Santé S.A.S., Lyon, France, an affiliate of Merck KGaA, Darmstadt, Germany at the time of the project. A. Granghaud was an employee of Pfizer S.A.S., Paris, France at the time of the study. J. Chang is an employee of Pfizer and holds stock and other ownership interest with Bayer, Bristol Myers Squibb, and Pfizer. M. Kearney is an employee of Merck KGaA, Darmstadt, Germany, and holds stock in Merck KGaA, Darmstadt, Germany, Novartis and UCB. A. Morel is an employee of Pfizer S.A.S., Paris, France. I. Plessala was an employee of Amaris Consulting, Paris, France at the time of the study. H. Cawston is an employee of Amaris Consulting, Paris, France. J. Roiz is an employee of and reposts stocks and other ownership interest with Evidera. Y. Xiao is an employee of Evidera. M.-N. Solbes is an employee of Merck Santé S.A.S., Lyon, France, an affiliate of Merck KGaA, Darmstadt, Germany. P. Lambert is an employee of Pfizer S.A.S., Paris, France. A. Ravaud has received grants or contracts from Merck KGaA, Darmstadt, Germany, and Pfizer; has received travel and accommodation expenses from Ipsen Merck KGaA, Darmstadt, Germany, Merck & Co., Kenilworth, NJ, and Pfizer; and has participated in advisory boards for Esai, Ipsen, Merck KGaA, Darmstadt, Germany, and Pfizer. Y. Loriot has served in consulting or advisory roles for Astellas Pharma, Bristol Myers Squibb, Immunomedics, Janssen, Loxo/Lilly, Merck KGaA, Darmstadt, Germany, Merck & Co., Kenilworth, NJ, Pfizer, Roche, Seattle Genetics, and Taiho Pharmaceutical; has received travel and accommodations expenses from Astellas Pharma, Janssen Oncology, Merck & Co., Kenilworth, NJ, Roche, and Seattle Genetics; and has received institutional research funding from Astellas Pharma, Basilea, Bristol Myers Squibb, Exelixis, Gilead Sciences, Incyte, Janssen Oncology, Merck KGaA, Darmstadt, Germany, Merck & Co., Kenilworth, NJ, Nektar, Pfizer, Roche, Sanofi, Seattle Genetics, and Taiho Pharmaceutical. A. Thiery-Vuillemin has participated in advisory boards for Astellas Pharma, AstraZeneca, Bristol-Myers Squibb, Ipsen, Janssen, Merck & Co., Kenilworth, NJ, Novartis, Pfizer, Roche/Genentech and Sanofi; reports employment by Bristol Myers Squibb; has served on steering committees for AstraZeneca, Bristol-Myers Squibb and Novartis; has received institutional research funding from Bayer, Ipsen and Pfizer; has served as principal investigator for Astellas Pharma, AstraZeneca, Bristol-Myers Squibb, Excelixis, Incyte, Ipsen, Johnson & Johnson, Merck & Co., Kenilworth, NJ, Novartis, Pfizer, Roche, Sanofi, and UNICANCER/GETUG; has received travel and accommodation expenses from Astellas Pharma, AstraZeneca, Bristol-Myers Squibb, Ipsen, Johnson & Johnson, Merck & Co., Kenilworth, NJ, Pfizer and Roche; and is a member of ASCO and GETUG. P. Lévy has served in consulting or advisory role and had received honoraria from Merck KGaA, Darmstadt, Germany.”  Please confirm that this does not alter your adherence to all PLOS ONE policies on sharing data and materials, by including the following statement: "This does not alter our adherence to PLOS ONE policies on sharing data and materials.” (as detailed online in our guide for authors http://journals.plos.org/plosone/s/competing-interests). If there are restrictions on sharing of data and/or materials, please state these. Please note that we cannot proceed with consideration of your article until this information has been declared.  Please include your updated Competing Interests statement in your cover letter; we will change the online submission form on your behalf. | The Competing Interests section has been updated to include the following statement:  “*This does not alter our adherence to PLOS ONE policies on sharing data and materials*.”  We have also updated the wording related to the funders in various places. |
| **5** | We note that you have indicated that data from this study are available upon request. PLOS only allows data to be available upon request if there are legal or ethical restrictions on sharing data publicly. For more information on unacceptable data access restrictions, please see http://journals.plos.org/plosone/s/data-availability#loc-unacceptable-data-access-restrictions.  In your revised cover letter, please address the following prompts:  a) If there are ethical or legal restrictions on sharing a de-identified data set, please explain them in detail (e.g., data contain potentially sensitive information, data are owned by a third-party organization, etc.) and who has imposed them (e.g., an ethics committee). Please also provide contact information for a data access committee, ethics committee, or other institutional body to which data requests may be sent.  b) If there are no restrictions, please upload the minimal anonymized data set necessary to replicate your study findings as either Supporting Information files or to a stable, public repository and provide us with the relevant URLs, DOIs, or accession numbers. For a list of acceptable repositories, please see http://journals.plos.org/plosone/s/data-availability#loc-recommended-repositories.  We will update your Data Availability statement on your behalf to reflect the information you provide. | Availability of data and material:  There are no restrictions in data availability. Previously published data from the phase 3 JAVELIN Bladder 100 trial were used in these analyses to inform efficacy, safety and health-related quality-of-life inputs. The trial results supporting these analyses are presented within the article and in the electronic supplementary material.  Patient-level data were not used in the analyses reported.  Code availability:  Analyses were performed using Excel (Microsoft Corporation) and Visual Basics for Applications. This model is not publicly available. |
| **6** | Please review your reference list to ensure that it is complete and correct. If you have cited papers that have been retracted, please include the rationale for doing so in the manuscript text, or remove these references and replace them with relevant current references. Any changes to the reference list should be mentioned in the rebuttal letter that accompanies your revised manuscript. If you need to cite a retracted article, indicate the article’s retracted status in the References list and also include a citation and full reference for the retraction notice. | The Reference list has been updated to ensure current relevant data sources are cited. Additionally, some hyperlinks were updated to ensure they work properly. |
| **No.** | **Editor comments** |  |
| **1** | This work is of high quality. However, I have a major concern about the availability of data. It should be included as a supplementary file. This work was conducted by researchers working for the pharmaceutical industry and therefore there is a potential conflict of interest. According to the explanation in the data sharing statement, the data will be shared only in specific cases. In terms of transparency, the data used for the analysis should be freely available. | We thank the editor for their feedback.  Previously published data from the phase 3 JAVELIN Bladder 100 trial were used in these analyses to inform efficacy, safety and health-related quality-of-life inputs. The trial results supporting these analyses are presented within the article and in the electronic supplementary material. Patient-level data were not used in the analyses reported.  Code availability:  Analyses were performed using Excel (Microsoft Corporation) and Visual Basics for Applications. This model is not publicly available. |
| **No** | **Reviewer #1 comments** |  |
| **1** | The article is a great piece of knowledge. Well written. A remarkable introduction and background, with well described methods and robust results. The article presented accurate graphs and conclusions are close related to the findings. | We thank the reviewer for their feedback. |
| **No** | **Reviewer #2 comments** |  |
| **1** | Dear Colleagues, thank you for the opportunity to review this interesting and well-written paper aiming to evaluate the cost-effectiveness of avelumab maintenance therapy plus BSC vs BSC alone for adults with locally advanced or metastatic urothelial carcinoma that had not progressed following platinum-based chemotherapy in France.  This type of analysis holds critical importance in informing decision makers and other stakeholders about the budgetary impact (not only cost) of innovative drugs, as well as serving as a tool to negotiate decreasing cost of expenditure when the results are not favorable.  The paper effectively and comprehensively outlines all the steps taken during the analysis. Although numerous adaptations are required to suit specific health systems, thresholds, costs, and legislations in other regions, it nonetheless offers a detailed guide that can be applied in various settings.  Having stated the above, I am afraid the extensive involvement of industry representatives can somehow undermine the credibility of the analysis. The participation of the scientific community in the validation of the model needs to be highlighted and explained in depth. | We thank the reviewer for their feedback. The involvement of the manufacturers of avelumab in the work reported is disclosed transparently in all relevant sections. As requested by the journal above, we have updated the funding statement to specify the role of the funders in the development of the manuscript:  *“Authors who were employees of the funders were involved in the model design, data collection, analysis, manuscript preparation, and the decision to publish.”*  As described in detail in the Introduction section, assessing the economic value of a new drug indication is a mandatory step of the French reimbursement process. The work described supported an application to the French National Authority for Health by Merck and Pfizer, thus extensive involvement of industry representatives is to be expected. The validation by the scientific committee including external experts is described in a specific paragraph in the Methods and in various places in the Discussion. |
