# Supplementary material for: Cost-effectiveness of avelumab first-line maintenance therapy for adult patients with locally advanced or metastatic urothelial carcinoma in France
Source: PLoS One. 2024 May 10;19(5):e0302548. doi: 10.1371/journal.pone.0302548 (PMC11086848; doi:10.1371/journal.pone.0302548)
Supplement: Attachment — Submitted filename: Response to Reviewers_France UC CE Manuscript_02APR24.docx [file pone.0302548.s002.docx]

**Response to reviewers’ comments**

**Manuscript title:** Cost-effectiveness of avelumab first-line maintenance therapy for adult patients with locally advanced or metastatic urothelial carcinoma in France

**Manuscript ID:** PONE-D-23-16035

**Authors:** Fanny Porte, Anna Granghaud, Jane Chang, Mairead Kearney, Aya Morel, Ingrid Plessala, Hélène Cawston, Julie Roiz, Ying Xiao, Marie-Noelle Solbes, Prisca Lambert, Alain Ravaud, Yohann Loriot, Antoine Thiery-Vuillemin, Pierre Lévy

| **No.** | **Journal Requirements** | **Response and proposed revisions (in red)** |
| --- | --- | --- |
| **1** | Please review your reference list to ensure that it is complete and correct. If you have cited papers that have been retracted, please include the rationale for doing so in the manuscript text, or remove these references and replace them with relevant current references. Any changes to the reference list should be mentioned in the rebuttal letter that accompanies your revised manuscript. If you need to cite a retracted article, indicate the article & retracted status in the References list and also include a citation and full reference for the retraction notice. | The reference list is complete and no retracted papers are cited. No changes are required. |
| **No.** | **Additional Editor Comments** | **Response and proposed revisions** |
| **1** | I guess all the comments given for the original manuscript is answered except for one.  In the comments for original submission, a reviewer has recommended that the raw data supporting the results of this study should be fully open to public. It is not shown whether the authors have any intention to do so in the R1 response.  If the authors choose not to make the database fully open, I have to consult another expert if this is appropriate.  *Previous response:*  *Availability of data and material:*  *There are no restrictions in data availability. Previously published data from the phase 3 JAVELIN Bladder 100 trial were used in these analyses to inform efficacy, safety and health-related quality-of-life inputs. The trial results supporting these analyses are presented within the article and in the electronic supplementary material.*  *Patient-level data were not used in the analyses reported.*  *Code availability:*  *Analyses were performed using Excel (Microsoft Corporation) and Visual Basics for Applications. This model is not publicly available.* | As noted in the previous response, the analysis was not performed using patient-level data, which means that no raw data were used for the analysis and no database was accessed. Analyses, such as Kaplan Meier curves, were based on previously published trial results, which are all presented within the article and supplementary material.  As also noted previously, the code used for the analyses is not publicly available, which is because it is proprietary.  Additionally, the Methods section was edited to reference the trial publication where the Kaplan Meier curves are reported. |
